# Missed Opportunity of Antenatal Care Services Utilization and Associated Factors among Reproductive Age Women in Eastern Hararghe Zone, Eastern Ethiopia: Mixed Methods Study

**DOI:** 10.1155/2023/8465463

**Published:** 2023-09-28

**Authors:** Ibsa Mussa, On Makhubela-Nkondo, Melat B. Maruta, Adera Debella

**Affiliations:** ^1^School of Public Health, College of Health and Medical Sciences, Haramaya University, Harar, Ethiopia; ^2^Department of Health Studies, College of Human Sciences, School of Social Sciences, University of South Africa, South Africa; ^3^Department of Obstetrics and Gynecology, Menelik Specialized Comprehensive Hospital, Addis Ababa, Ethiopia; ^4^School of Nursing and Midwifery, College of Health and Medical Sciences, Haramaya University, Harar, Ethiopia

## Abstract

**Background:**

Despite the enormous advantages of early pregnancy-related problem diagnosis and therapy during prenatal care visits, not all pregnant women begin antenatal care at the proper time. Thus, this study aims to identify factors associated with missed opportunities for antenatal care service utilization among reproductive-age women in Eastern Ethiopia.

**Methods:**

A mixed methods study design (quantitative and qualitative) was conducted in Grawa, Meta, and Haramaya woredas from September 5 to December 5, 2019. The quantitative data were analyzed using SPSS version 25. A multivariable logistic regression analysis model was used to identify the predictors. Statistical software programs based on ATLAS.ti version 8.2 was were used to conduct the thematic analysis of the qualitative data.

**Results:**

Overall, missed opportunities for antenatal care were 15.4% of 95% (12.1, 19.1%). Factors such as maternal age being 15–24 (AOR = 6.9, 95% CI: 2.89–8.81); having a college education (AOR = 0.02, 95% CI: 0.001, 0.42), elementary (AOR = 0.05, 95% CI: 0.002, 0.98), and secondary education (AOR = 0.04, 95% CI: 0.001, 0.88); having five and more parity (AOR = 0.08, 95% CI: 0.01, 0.75); three visits (AOR = 0.10, 95% CI: 0.02, 0.71); those in the first trimester (AOR = 0.02, 95% CI: 0.001, 0.35) and the second trimester (AOR = 0.01, 95% CI: 0.001, 0.26); and get information from a health facility (AOR =0.09, 95% CI: 0.01, 0.67) and traditional birth attendance (AOR = 0.02, 95% CI: 0.001, 0.74) were factors statistically associated with outcome variables.

**Conclusions:**

According to this report, relatively high proportions of pregnant women experienced missed opportunities in antenatal care follow-up. Factors such as maternal age, education, parity, frequency, timing, and media access were statistically significantly correlated with missed antenatal care follow-up. Therefore, all stakeholders should emphasize advocating for and enhancing the benefits of antenatal care; this in turn plays a crucial role in increasing the follow-up of clients for these crucial services. Moreover, health policy implementers need to coordinate their tracking of pregnant women who missed their antenatal care session.

## 1. Introduction

Globally, an estimated 303 million women died from complications related to pregnancy, childbirth, and postpartum period [1, 2]. Every day, about 830 pregnant women pass away from curable or preventable pregnancy-related conditions. Among these, ninety-nine percent (822) of women die in developing countries due to pregnancy and childbirth [3]. Even though maternal mortality is a global issue, its major issues are most felt in underdeveloped countries. As a result, developing nations account for 99 percent of the estimated global number of maternal fatalities, with an estimated 201,000 of those deaths happening in sub-Saharan Africa [4].

Quality antenatal care, accessible obstetrics care, and life-saving interventions are critical services for reducing preventable maternal and newborn mortality and morbidity [5–7]. Bringing the global maternal death rate down to fewer than 70 per 100,000 live births is a key component of Sustainable Development Goal (SDG) 3.1. [8]. To eliminate preventable causes of maternal death due to pregnancy-related complications, existing interventions during pregnancy and delivery must be scaled up [7]. Hemorrhage, hypertension, and infection are the leading causes of maternal death [9, 10]. The most common causes of newborn deaths and stillbirths are asphyxia, prematurity, and infections, and the majority are preventable with existing evidence-based interventions, either directly or indirectly, during pregnancy, labor, delivery, and postpartum [5, 7, 11, 12].

Despite the fact that universal coverage of antenatal care services is reported globally and at the country level, many pregnant women in developing countries do not benefit from comprehensive antenatal care due to factors such as late initiation and poor quality of antenatal care services [13–16]. Early initiation during the first trimester and quality antenatal care throughout the pregnancy have been shown to improve pregnancy outcomes and increase newborn survival [17]. However, in developing countries, only 25% of pregnant women initiated antenatal care before 14 weeks of gestation, and 48% of pregnant women did not complete four antenatal care visits [14].

Despite the disparities in maternal death rates across developed and developing countries, studies have demonstrated that the pattern of maternal mortality and morbidity has remained constant throughout time. The reasons given for this include the long-standing custom of having births at home in risky and unclean settings with untrained or inadequately qualified delivery attendants [5, 18, 19].

In Ethiopia, the maternal death ratio decreased from 743/100,000 new births in 2005 to 353/100,000 in 2015. This puts Ethiopian women at a much higher risk than their counterparts in the developed world, where the risk was estimated to be 1 in 23,700 and 1 in 22,100 in nations like Greece and Poland, respectively. As a result, the likelihood of an Ethiopian woman dying from reproductive health disorders and complications was put at 1 in 25 in 2005, 1 in 39 in 2010, and 1 in 64 in 2015 [3, 18].

Despite the government's and numerous stakeholders' dedication to advocating for and enhancing the benefits of antenatal care, the prevalence of missed opportunities and factors influencing them remain unabated. Additionally, to researchers' knowledge, there is a dearth of published material pertaining to missed opportunities and their related causes in the study region. Furthermore, there is no single study that employed qualitative methods to dig out the variable under study. As a result, this study assessed the prevalence of missed opportunities and associated factors in the study area.

## 2. Methods and Materials

### 2.1. Study Design, Setting, and Period

**Figure 1:**
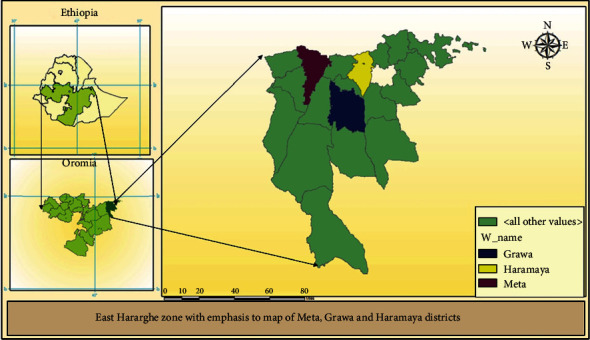
Map showing of study setting, namely, Haramaya, Meta, and Grawa, 2019.

A mixed methods study (cross-sectional quantitative method and phenomenology qualitative method) was conducted from September 5, 2019, to December 5, 2019, among 422 pregnant women in Grawa, Meta, and Haramaya woredas. This study site, namely, Haramaya, Meta, and Grawa, is situated in Eastern Ethiopia, 526, 423, and 552 kilometers from the capital city of Ethiopia, Addis Ababa. According to data from the woreda health office in 2015, the populations of the districts were 291,000, 226,000, and 313,000. Of these, reproductive age groups account for a total population of 124,470. Most residents make their living through farming and small-scale trading (Figure 1).

### 2.2. Population and Eligibility Criteria

All woreda-dwelling women of reproductive age who had given birth within the previous five years of the data gathering were considered the source population, whereas randomly selected women using the sampling technique who were available during the data collection period were regarded as the study population. The inclusion criteria were women who were permanent residents (who lived more than one year in the study area) in the selected districts and women whose ages were between 15 and 49 years. Women with hearing impairments or communication impairment conditions, as well as those who were critically ill, had mental health issues, or had been alive for less than a year at the time of the interview, were excluded.

### 2.3. Sample Size Determination and Sampling Procedures

The sampling size was computed by using a single population ratio formula based on the assumption that to get the largest sample size possible and in light of the lack of prior prevalence statistics on the population being studied, *p* was taken to be 0.5. Additionally, a 95% confidence interval and a 5% margin of error were established (*Z*/2 = 1.96) [20], women aged between 15 and 49 years made up the majority of the total population (*N* = 124,470), and a sample size of 422 women was determined using a 10% contingency for nonresponse [21].

A three-stage sampling approach was used in this investigation. 30% of the total kebeles in each district were included in the initial stage. As the number of kebeles varies in the three districts, proportional sampling to give equal weights was used [22]. As a result, samples were taken from 39 percent of kebeles in Grawa, 33 percent in Meta, and 28 percent in Haramaya [18, 19]. Based on the size of the population, 10 kebeles and 118 HHs from Haramaya, 12 kebeles and 165 HHs from Meta, and 14 kebeles and 139 HHs from Grawa were selected for the sample of kebeles and homes (HHS). A lottery technique was used to choose the respondents, with villages being selected at random [23]. Last but not least, research subjects took samples from kebeles in the three woredas; a total of 422 study samples were chosen utilizing a systematic sampling technique. Then, each patient was given a unique code.

The sample size determination and sampling procedures for antenatal care service utilization and associated factors for qualitative data included 8 focus group discussants and 32 respondents in a homogenous group (i.e., 4 community health workers, 8 women in reproductive age, 4 husbands, 4 religious leaders, and 4 community representatives). Additionally, five mothers-in-law, two sisters, and a father-in-law of the eight relatives who participated in decision-making during the birthing process were interviewed [24].

### 2.4. Data Collection Methods

The open-ended questionnaire, in-depth interviews, audiovisual materials, focus group discussions, and field observation were used to gather qualitative information, while an interviewer-administered, pretested closed-ended structured questionnaire, instruments, behavioral checklists, and records were used to gather quantitative information [25]. Representatives from the district health office, health center, and women's affairs participated in in-depth interviews [26]. Using previously developed interview criteria, focus groups were done with women of reproductive age, community and religious leaders, spouses, and mothers-in-law [22]. It contains sociodemographic and economic characteristics, household-related conditions, healthcare services, and comorbidity-related characteristics.

Sixteen female data collectors with a minimum of secondary education and native Afaan Oromo speakers gathered the data. The principal investigator was supervised by two public health professionals who are familiar with the study's environment. Three days of training on ethics, tools, sampling, and data collection techniques was given to data collectors and supervisors. Daily oversight of the data gathering process was provided, and the data collectors received prompt feedback [18, 19].

### 2.5. Variables and Their Measurement

*Antenatal care (ANC)* is the proportion of women aged 15 to 49 who gave birth alive and got prenatal care four or more times within a specific time period [5].

*Missed opportunities* in maternal and infant health research are built on the premise that considerable chances for enhancing mother and child health have been missed or might have been avoided and that a better arrangement and focus on postpartum care will lower maternal morbidity and death [27].

A traditional birth attendant (TBA) is a person who assists a mother during prenatal and postpartum care and who initially acquired her skills by delivering babies herself or through apprenticeship to other traditional birth attendants [18, 19, 28].

### 2.6. Data Quality Control

A bilingual expert (Afaan Oromo) created the questionnaire in English before translating it into the regional tongues. To guarantee uniformity, it was then translated back into an English version. The supervisor and data collectors both got training on the methods and tools for collecting data. 10% of the study participants took a pretest in a setting similar to research data collection. Regular oversight was given by the investigators and skilled research supervisors.

### 2.7. Data Processing and Analysis

The acquired information was initially checked for consistency and completeness. Then, for further analysis, they were cleaned, coded, and put to EpiData version 3.1. The entered data were transferred to SPSS version 25 for analysis. Descriptive and summary data were produced and published using frequency tables and figures. A binary logistic regression model was created to examine any connections between the independent variables and the outcome variable. The fitness of the model was assessed by Hosmer-Lemeshow statistics and Omnibus testing. A multivariable analysis was used to identify the real factors that affected the result variables. To determine whether there was any relationship between the independent variables, a multicollinearity test was carried out using the standard error and colinearity statistics. There were no detectable collinearity effects. With this in mind, the variance inflation factor (VIF) was 0.951. To ascertain the strength and direction of the statistical association, the odds ratio (OR) and 95% confidence interval (CI) were utilized. At a 0.05 *p* value, it was determined that both the bivariate and multivariate analyses were statistically significant.

Atlas.ti version 8.2 is was employed to assess qualitative data. Theme analysis was used to complete it, analyzing and interpreting the collected data. After then, other codes were used to encode the translated data. Each code was split up into a variety of groupings, which were then grouped into themes.

## 3. Results

### 3.1. Socioeconomic and Demographic Characteristics

A total of 422 study participants between the ages of 15 and 49 participated, with a 100% response rate. The mean age of participants in the research was 26 years old, with SD ± 6.3. The majority of the study subjects were married: 324 (76.8%), and 225 (53.3%) were unable to read and write, whereas husbands were 238 (56.4%) in elementary school. Of almost all of the participants, three hundred eighty-nine (92.2%) were Oromo ethnic group, and 385 (91.2%) were Muslims. Almost 101 (23.9%) of the respondents had a household family size of one to four (Table 1).

### 3.2. Pregnancy-, Cultural-, and Behavioral-Related Characteristics

The mean (±SD) age of the first marriage and first pregnancy was 25.7 ± (5.7) and 27.4 ± (5.8), respectively. The majority of study participants, 166 (39.3%), had two to four children, and more than half, 223 (52.8%), were in the age range between 25 and 34 years. Regarding adverse pregnancy outcomes, approximately 393 (93.1%) and 391 (92.7%) women had a previous history of abortion and stillbirth, respectively. More than half of the 290 participants (68.7%) were not planning a pregnancy, and 206 (48.8%) had a history of female genital mutilation. Regarding decision-making authority, 342 (81.1%) made a joint decision, while the majority of the respondents 372 (88.2%) of the total study participants had experienced intimate partner violence, whereas 248 (58.8%) of the respondents had not received support from their partners (Table 2).

### 3.3. Proportion of Missed Opportunity of Antenatal Care

**Figure 2:**
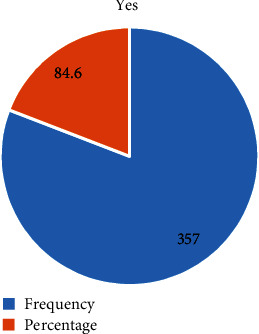
The prevalence of missed opportunity of ANC services among reproductive age women in Eastern Ethiopia, 2019.

Among 422 study participants, 357 (84.6%) had antenatal care (ANC) follow-up whereas 65 (15.4%) missed ANC follow-up (Figure 2).

### 3.4. Obstetrics and Health Service-Related Variables

The majority of the women, 357 (84.6%), visited health facilities at least once for prenatal care, and from the total of 422 responders, 202 (56.6%) made their first booking in the first trimester, while the remaining participants initiated antenatal care (ANC) in the second or third trimester. Additionally, 204 participants (57.1%) had only used ANC services three times, while 233 respondents (65.3%) received information about ANC from the health institution, 200 respondents (56.0%) had to wait between 31 and 60 minutes for services, and 217 respondents (60.7%) were shown respect by the medical staff. The majority of the women, 205 of the respondents (48.6%), did not have access to convenient transportation from their homes to the nearest health facility, while 221 respondents (52.4%) received ANC service from the health center. Three-fourths of the 268 respondents (75.1%) had started their ANC services because of health problems. Nonetheless, 208 (58.3%) of the study's participants lack confidence in medical professionals. The survey discovered that 266 respondents (74.5%) had received health education while getting ANC. Regarding privacy, more than 294 respondents (82.4%) stated that their privacy was not respected during ANC visits (Table 3).

Moreover, the focus group discussion (FGD) participants strengthened the ideas provided above, and one, a 25-year-old female focus group discussant, expressed the difficulty of maintaining privacy and confidentiality. She stated that

*the male doctor inserted his fingers into my vagina, and other nurses stood there looking at him. Perhaps they were in training. I feel bad seeing that crowd staring at me.... Because there were no curtains and no private room, it was difficult to change my clothes and breastfeed the baby after birth. We can see each other, and others (visitors) can easily see us.... After the baby is born, one bed for each woman and private rooms or curtains around the bed to avoid seeing other women would be more comfortable for all women.*

In a similar vein, one participant in FGD said that

*when I went to the hospital for an antenatal check-up, there was a health care provider.... It was not what I had hoped for before going to the hospital for service. There was no respectful healthcare provider.... The male doctors were there to perform examinations. The doctors pressed hard on my tummy, causing me pain.*

Thus, from the above discussion, we conclude that the way the healthcare providers treat the woman plays a pivotal role in keeping her on track with ANC.

Furthermore, one of the participants immensely complained about the waiting time in the ANC and said that

*we are busy at home with our daily tasks …..and when we go for ANC visits, we are expected to stay at least an hour. We have a lot of daily activities that we do during this time. As a result, we are clearly tired of waiting, which leads us to abandon follow-up. (A 29-year-old woman)*

**Figure 3:**
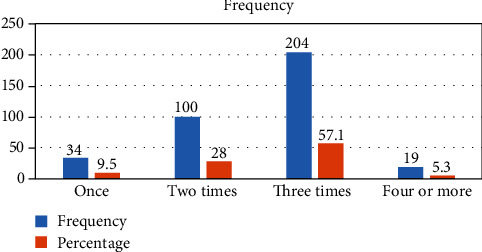
The frequency of missed opportunity of ANC services among reproductive age women in Eastern Ethiopia, 2019.

Among the total 422 study participants, 204 (57.1%) (95% CI: 52.0–62.3) were pregnant women attending ANC three times, while 19 (5.3%) (95% CI: 3.1–7.8) attended ANC four or more times (Figure 3).

### 3.5. Missed Opportunity and Associated Factors

In the bivariable analysis, predictor variables such as maternal age, educational status, occupational status, family size, age at first marriage, parity, number of stillbirths, intention to conceive, frequency of ANC visits, timing of ANC, and source of information about ANC were significantly associated with antenatal care service utilization. However, in the final model of multivariable logistic regression analysis, predictor variables like those women in the age range of 15–24 years, who are unable to read and write, elementary and secondary education status, parity one, have three round visits, being in the first and second trimesters, and those who heard information from radio, TV, and TBA were significantly associated with ANC utilization.

Accordingly, respondents in the age range of 15–24 years were 6.9 times more likely to miss the opportunity of ANC than those women in the age range of 35–49 years (AOR = 6.90, 95% CI: 2.89, 8.81). Likewise, women who were in college and in elementary and secondary education were 98.1%, 95.2%, and 96.5% less likely to miss the ANC service than those who had no education or were unable to read and write (AOR = 0.019, 95% CI: 0.001, 0.421; AOR = 0.048, 95% CI: 0.002, 0.984; and AOR = 0.035, 95% CI: 0.001, 0.882, respectively). Moreover, those study participants who had more than five parities were 92% less likely to miss ANC services as compared to those who had one parity (AOR = 0.081, 95% CI: 0.009, 0.751). Likewise, those study participants who had three or more visits to ANC were 89.7% less likely to miss ANC services than those who had one visit (AOR = 0.103, 95% CI: 0.015, 0.711).

Furthermore, the odds of missing ANC services for pregnant women who came for ANC follow-up during the first and second trimesters were 98% and 99% less likely as compared to their counterparts (AOR = 0.020, 95% CI: 0.001, 0.346, and AOR = 0.012, 95% CI: 0.001, 0.260, respectively). Likewise, the study participants who heard information from TV, radio, and TBA were 91% and 98% less likely to miss ANC services than those who heard from other sources (AOR = 0.092, 95% CI: 0.013, 0.671, and AOR = 0.022, 95% CI: 0.001, 0.743) (Table 4).

## 4. Discussions

This study assessed the missed opportunity of antenatal care services and associated factors among reproductive age women in Haramaya, Chelenko, and Grawa districts, Eastern Ethiopia. This study pointed out that 15.4% (95% CI: 12.1%, 19.1%) of study participants missed out on antenatal care services. Thus, more than four out of every five pregnant women utilized antenatal care services. Factors such as those who are women in the age range of 15–24 years, unable to read and write, elementary and secondary education status, those who have one parity, those who have three round visits, being in the first and second trimesters, those who heard information from a health care provider, and TBA were identified as predictors of ANC utilization.

According to this study, 15.4% of study participants missed the opportunity for antenatal care services. This is in harmony with a study conducted in different settings, such as Kenya (12%) and Malawi (15%) [29]; Holeta Town, Central Ethiopia (13%) [24]; Northwest Tanzania (13%) [30]; Tigray Region (18%) [31]; and Hadiya Zone (14%) [32]. However, the prevalence of missed opportunities in the current study was much lower than the previous studies conducted in different settings like Gelemso (34.6%) [33], Northern Tigray (33.4%) [34], EDHS (68%) [35], Ethiopia (25.7) [36], Southern Ethiopia (23.8) [37], Northwest Ethiopia (56.0) [38], China (19.7%) [39], Afghanistan (30.7%) [40], and Addis Ababa (22.4%) [41]. Moreover, according to the 2011 Ethiopian Demographic and Health Survey (EDHS), 76% of women in urban areas used ANC. The comparative figures for rural areas and the national level were 26% and 34%, respectively [42]. The possible justification for these disparities might be attributed to differences in sample size, methods of assessment, and the time gaps of the study period. Another possible explanation is that the study population has lower access to information on maternal healthcare services, of which ANC is one of the vital components. In contrast to this, the findings from this study are relatively higher than those from studies conducted in different settings, such as Jimma Town (10%) [43]. The possible difference may be due to the fact that East Ethiopia has a higher level of ANC use, which may be due to the fact that the area was a cash crop area of Ethiopia and could have better opportunities for information and better access to health institutions than the other areas. In addition, seasonal variability when the studies were conducted could explain the observed difference among the findings.

Findings from this study pointed out that the age of women was a significant predictor of outcome variables. Thus, women in the age range of 15–24 were 6.90 times more likely to miss ANC opportunities than women in the age range of 35 and above. This is consistent with the study conducted in Kenya, Malawi, and Ghana [29]. It is crystal clear that this cohort of the population is youth and adolescents. Hence, much has to be expected to enhance their concerns and complaints related to those of the ANC, which in turn plays a crucial role in reversing their absence from ANC services. Furthermore, this is partially explained by the fact that women in age groups above 35 were more adapted to and integrated into the services than their counterparts.

The results of our research demonstrate that ANC services were used more frequently as maternal education levels increased. This result is conceivable because increased education will give women a greater understanding of and awareness of ANC services. Furthermore, self-independence, confident decision-making, and capability for managing household and health all increased with better educational levels [44, 45]. Findings show that respondents who were unable to read and write in elementary and secondary education were 98.1%, 95.2%, and 96.5% more likely to miss ANC service than those who had college and above. The odds of missing an antenatal care service were reduced when educational status increased. This finding was supported by previous research in Southern Ethiopia [43], Northwest Ethiopia [46, 47], and Western Ethiopia [48]; a systematic review conducted in Ethiopia [49]; and EDS carried out in rural Ethiopia [35, 50], Kenya [51], Guinea [52, 53], Afghanistan [40], Angola [54], East African countries [55], India [56, 57], Nepal [58], and Nigeria [59]. Educated women might be empowered to get services [60–65], and education makes women have decision-making skills [65, 66]. On top of this, educated women have knowledge of danger signs [66–68]. Educated women might be aware of the advantages of antenatal care services provided at the service delivery points for the fetus and themselves [34, 69, 70]. Moreover, this may be linked to the fact that more educated women are aware of the advantages of using ANC, which enhances maternal and fetal health through early detection of issues connected to pregnancy and the receipt of different preventative and health promotion services. Also, educated women can have access to media outlets that promote the importance of prompt ANC commencement and healthcare facilities that offer the treatment. Contrary to these, illiterate women could not fully appreciate the value of ANC services for the health and safety of their unborn fetus because of the lack of knowledge about the pros and cons of ANC.

The likelihood of utilization of ANC services among women who had five or more births was 92% less likely to be missed compared to women who had one birth. This study is supported by studies conducted in Eastern Ethiopia [71] and elsewhere in Cambodia [72]. One explanation for this could be that first-time mothers are more likely than second- or third-time mothers to be afraid of getting pregnant and want to deliver their babies in a hospital [73]. A study showed that women perceive that the first pregnancy could bring more risks than subsequent pregnancies, and they become less attentive when the number of pregnancies exceeds a certain limit [74]. Furthermore, women who had a history of giving birth safely would feel they were healthy and not intend to seek delivery services from health facilities [75]. In addition, evidence indicates that women who have many children are less likely to give birth in a healthy institution [76]. Therefore, community health programs should consider community awareness creation to improve institutional delivery among women with multipara.

According to the WHO, every expectant mother should go to at least four antenatal care appointments [5] and give birth at a health facility with the assistance of a skilled healthcare provider [35]. According to our study, women who had three ANC visits were 89.7% less likely to miss ANC services than who had only one ANC visit. Our findings were consistent with the research done in Eastern Hararghe [77]. However, it was less than the results of research carried out in the Tigray Region [78] and Dembecha District [79] in Ethiopia, as well as in Nigeria [80]. The probable variations in the supply of high-quality healthcare services and limited access to transportation could be the likely causes of the differences.

This research showed that optimal ANC was better as compared to the findings of previous studies done in Nekmet, Ethiopia (32.1%) [81], and elsewhere in Kenya (32%) [82]. The reason for this variation could be the emphasis given by the government of Ethiopia to improve maternal health service utilization, considering that it is one of the exempted services in Ethiopia [83].

Furthermore, this study pointed out that pregnant women who came for ANC follow-up in the fourth and second trimesters were found to be important predictors of outcome variables. Thus, pregnant women who came for ANC follow-up during the fourth and second trimesters were 98% and 99% less likely to miss out on ANC services as compared to their counterparts, respectively. Studies carried out in many contexts, including Gonder, Northwest Ethiopia, provide support for this study [84], Addis Ababa [41], and Malawi [85]. The possible justification could be the fact that pregnant women who came to ANC follow-up during the second and third trimesters may hugely understand the benefit of ANC as they are subjected to the thought that they missed plenty of benefits provided to pregnant women. This regression clearly enhances and tightens the need to be actively involved in the services of the ANC. Furthermore, because they joined this vital service late in the pregnancy journey and after experiencing the vital benefits of ANC services, they continue to use it.

Finally, in this study, the source of information for ANC services was found to be significantly associated with outcome variables. Hence, the study participants who heard information from TV, radio, and relatives were 95.4% and 97.9% less likely to use ANC services, respectively. This is congruent with the results of EDHS conducted in 2016 [86], Amhara [87], Holeta [24], and Addis Ababa [41]. Although a relative or a partner may be a trusted source of information, there is a chance of the dissemination of biased information, which in turn affects the utilization of antenatal care. Moreover, nowadays, people are fed up with mainstream media, which leads them not to attend the media as they think that the information disseminated through them is not reliable.

## 5. Practical Implications and Future Research Direction

This investigation made it clear that some of those pregnant women lost the chance to receive ANC. Therefore, people who successfully and efficiently received concentrated ANC visits profited considerably and immensely from the advice and assistance of healthcare professionals working in the setup. More significantly, it gave the women practical knowledge about birth preparation, preparing for problems, early treatment, and spotting concurrent issues. We strongly urge researchers to employ observational studies to ascertain the impacts of the missed chance of ANC on the health of mothers and their babies since we are only worried about the missed opportunity of ANC rather than its result. Furthermore, because the study was conducted at the community level, extrapolating results for those who worked in institutions would be difficult, which in turn affected the external validity of the study.

## 6. Limitation

The limitation of this study is that it does not address the qualitative aspects, like the detailed reasons why mothers missed their antenatal care follow-up. Therefore, it is open for researchers to conduct a qualitative study on why women missed their subsequent antenatal care follow-up.

## 7. Conclusions

This study pointed out that relatively high proportions of pregnant women experienced missed opportunities in antenatal care follow-up. Factors such as those who are women in the age range of 15–24 years, unable to read and write, elementary and secondary education status, those who have one parity, those who have three round visits, being in the first and second trimesters, those who heard information from a health care provider, and TBA were identified as predictors of ANC utilization. Therefore, in order to enhance the uptake of four full visits, health policy implementers need to coordinate their tracking of pregnant women who missed their antenatal care session.

## Figures and Tables

**Table 1:** Sociodemographic and economic characteristics of study participants, Eastern Ethiopia, 2019.

Variables (*n* = 422)	Categories	Frequency	Percentage
Maternal age	15–24 years	196	46.4
	25–34 years	161	38.2
	35-49 years	65	15.4

Educational status	Unable to read and write	225	53.3
	Elementary	123	29.2
	Secondary	63	14.9
	Collage and above	11	2.6

Occupational status	Government employee	69	16.4
	Self-employee/farmer	211	50.0
	Merchant	63	14.9
	Student	79	18.7

Current marital status	Married	324	76.8
	Single	5	1.2
	Divorced	52	12.3
	Widowed	29	6.9
	Separated	12	2.8

Ethnicity	Oromo	389	92.2
	Amhara	30	7.1
	Somali	3	0.7

Religion	Muslim	385	91.2
	Christian	32	7.6
	Waqefata	5	1.2

Educational status of husband	Unable to read and write	39	9.2
	Elementary	238	56.4
	Secondary	94	22.3
	Collage/university	51	12.1

Husband's occupation	Employee	69	16.4
	Self-employee/farmers	211	50.0
	Merchant	63	14.9
	Student	79	18.7

Family size	1-4	101	23.9
	5-8	202	47.9
	Above 9	119	28.2

**Table 2:** Pregnancy-, cultural-, and behavioral-related characteristics of study participants, Eastern Ethiopia, 2019.

Variables (*n* = 422)	Categories	Frequency	Percentage
Age at first marriage	15-25	197	46.7
	25-34	189	44.8
	35-49	36	8.5

Age at first pregnancy	15-25	145	34.4
	25-34	223	52.8
	35-49	54	12.8

Parity	1	110	26.1
	2-4	166	39.3
	≥5	146	34.6

Number of alive children	1	45	10.7
	2-3	230	54.5
	4-5	121	28.7
	≥6	26	6.1

Number of still birth	0	393	93.1
	1-2	29	6.9

History of abortion	No	391	92.7
	Yes	31	7.3

Type of abortion	Induced	7	22.6
	Spontaneous	24	77.4

Intention to pregnancy	Unplanned	290	68.7
	Planned	132	31.3

Previous history of female genital mutation	No	216	51.2
	Yes	206	48.8

Polygamy	One	400	94.8
	Two	19	4.5
	Three	3	0.7

Autonomy of mothers in decision-making	Wife	6	1.4
	Husband	74	17.5
	Joint	342	81.1

Intimate violence	No	50	11.8
	Yes	372	88.2

Husband support	No	248	58.8
	Yes	174	41.2

**Table 3:** Obstetrics and health service-related variables of study participants, Eastern Ethiopia, 2019.

Variables (*n* = 422)	Categories	Frequency	Percentage
ANC follow-up	Yes	357	84.6
	No	65	15.4

Timing of ANC	First trimester	202	56.6
	Second trimester	124	34.7
	Third trimester	31	8.7

Source of information about ANC	Health institution	233	65.3
	Radio/TV	31	8.7
	TBA	20	5.6
	Relatives	38	10.6
	Community health agent	19	5.3
	Friends	16	4.5

Did you receive a respect from healthcare provider	Yes	217	60.8
	No	140	39.2

Waiting time to get ANC (in minutes)	<30 min	118	33.1
	31-60 min	200	56.0
	>61 min	39	10.9

Distance to the nearest health institution	<30 min walking	91	25.5
	31-60 min walking	126	35.3
	>60 min walking	140	39.2

Easy transport service from home to the nearest health facility	Yes	217	51.4
	No	205	48.6

From where you receive your ANC	Hospital	86	20.4
	Health center	221	52.4
	Health post	115	27.2

Reason for initiating ANC	Health problems	268	75.1
	Start regular checkup	85	23.8
	Other^∗∗^	4	1.1

Health education during ANC	No	86	24.1
	Yes	266	74.5
	I do not know	5	1.4

Lack of privacy during ANC	Yes	294	82.4
	No	63	17.6

Confidence on health service	Yes	146	40.9
	No	208	58.3
	I do not know	3	0.8

^∗∗^Relatives and family interest.

**Table 4:** Bivariable and multivariable logistic regression analyses of factors associated with utilization of ANC care service utilization in Eastern Ethiopia, 2019.

Factors	Categories	Missed ANC utilization		COR (95% CI)	AOR (95% CI)
		Yes (%)	No (%)		
Maternal age	15–24 years	147 (41.2)	49 (75.4)	2.76 (1.183-6.451)	6.90 (2.89-8.81)^∗∗^
	25–34 years	152 (42.6)	9 (13.8)	0.491 (0.175-1.378)	4.72 (0.414-53.826)
	35-49 years	58 (16.2)	7 (10.8)	1	1

Educational status of mother	Unable to read and write	193 (54.1)	32 (49.2)	1	1
	Elementary	105 (29.4)	18 (27.7)	0.300 (0.080-1.130)	0.05 (0.002-0.984)^∗^
	Secondary	52 (14.6)	11 (16.9)	0.370 (0.092-1.486)	0.03 (0.001-0.882)^∗^
	Collage and above	7 (2.0)	4 (6.2)	0.290 (0.080-1.048)	0.02 (0.001-0.421)^∗^

Occupational status	Employee (gov't/NGO)	62 (17.4)	7 (10.8)	0.383 (0.149-0.981)	0.20 (0.036-1.153)
	Self-employee/farmers	180 (50.4)	31 (47.7)	0.584 (0.305-1.117)	0.43 (0.113-1.666)
	Merchant	54 (15.1)	9 (13.8)	0.565 (0.234-1.362)	0.90 (0.176-4.643)
	Student	61 (17.1)	18 (27.7)	1	1

Family size	1-4	78 (21.9)	23 (35.4)	2.044 (1.001-4.174)	0.14 (0.015-1.31)
	5-8	175 (49.0)	27 (41.5)	1.070 (0.544-2.104)	0.72 (0.165-3.173)
	≥9	104 (29.1)	15 (23.1)	1	1

Age at first marriage	15-25	169 (47.4)	28 (43.1)	0.580 (0.240-1.400)	2.48 (0.203-30.392)
	25-34	160 (44.8)	29 (44.6)	0.634 (0.263-1.529)	2.61 (0.217-31.341)
	35-49	28 (7.8)	8 (12.3)	1	1

Parity	1	97 (27.2)	13 (20.0)	1	1
	2-4	147 (41.2)	19 (29.2)	0.443 (0.239-0.819)	0.74 (0.164-3.407)
	≥5	113 (31.6)	33 (50.8)	0.459 (0.229-0.921)	0.08 (0.009-0.751)^∗^

Number of still birth	0	340 (95.2)	53 (81.5)	0.221 (0.100-0.488)	0.18 (0.029-1. 204)
	1-2	17 (4.8)	12(18.5)	1	1

Intention to pregnancy	Unplanned	244 (68.3)	46 (70.8)	1.121 (0.628-2.001)	3.22 (1. 846-12.286)
	Planned	113 (31.7)	19 (29.2)	1	1

Frequency of ANC visits	Once	27 (9.1)	7 (11.9)	1	1
	2 times	93 (31.2)	7 (11.9)	0.163 (0.047-0.561)	0.58 (0.065-5.307)
	3 times	165 (55.4)	39 (66.1)	0.359 (0.126-1.020)	0.10 (0.015-0.711)^∗^
	4 or more	13 (4.3)	6 (10.1)	0.562 (0.157-2.011)	1.97 (0.152-25.618)

Timing of ANC	First trimester	182 (57.1)	20 (52.6)	0.152 (0.065-0.356)	0.02 (0.001-0.346)^∗∗^
	Second trimester	119 (37.3)	5 (13.2)	0.058 (0.019-0.183)	0.01 (0.001-0.260)^∗∗^
	Third trimester	18 (5.6)	13 (34.2)	1	1

Source of information about ANC	Health institution	207 (67.0)	26 (54.2)	0.276 (0.089-0.858)	0.09 (0.013-0.671)^∗^
	Radio/TV	24 (7.8)	7 (14.6)	0.642 (0.166-2.479)	0.46 (0.028-7.530)
	TBA	14 (4.5)	6 (12.5)	0.943 (0.227-3.922)	0.02 (0.001-0.743)^∗^
	Relatives	35 (11.3)	3 (6.2)	0.189 (0.039-0.919)	0.23 (0.019-2.855)
	CHA	18 (5.8)	1 (2.1)	0.122 (0.013-1.188)	0.20 (0.003-15.663)
	Friends	11 (3.6)	5 (10.4)	1	1

^∗^ *p* value < 0.01; ^∗∗^*p* < 0.05.

## Data Availability

The data sets used for this study are available from the corresponding author on reasonable request.
